# Transforming experiences: Neurobiology of memory updating/editing

**DOI:** 10.3389/fnsys.2023.1103770

**Published:** 2023-02-21

**Authors:** Daniel Osorio-Gómez, Maria Isabel Miranda, Kioko Guzmán-Ramos, Federico Bermúdez-Rattoni

**Affiliations:** ^1^División de Neurociencias, Instituto de Fisiología Celular, Universidad Nacional Autónoma de México, Mexico City, Mexico; ^2^Departamento de Neurobiología Conductual y Cognitiva, Instituto de Neurobiología, Universidad Nacional Autónoma de México, Juriquilla, Mexico; ^3^División de Ciencias Biológicas y de la Salud, Departamento de Ciencias de la Salud, Universidad Autónoma Metropolitana, Lerma de Villada, Mexico

**Keywords:** recognition memory, associative learning, valence shifting, novelty and familiarity, reconsolidation

## Abstract

Long-term memory is achieved through a consolidation process where structural and molecular changes integrate information into a stable memory. However, environmental conditions constantly change, and organisms must adapt their behavior by updating their memories, providing dynamic flexibility for adaptive responses. Consequently, novel stimulation/experiences can be integrated during memory retrieval; where consolidated memories are updated by a dynamic process after the appearance of a prediction error or by the exposure to new information, generating edited memories. This review will discuss the neurobiological systems involved in memory updating including recognition memory and emotional memories. In this regard, we will review the salient and emotional experiences that promote the gradual shifting from displeasure to pleasure (or vice versa), leading to hedonic or aversive responses, throughout memory updating. Finally, we will discuss evidence regarding memory updating and its potential clinical implication in drug addiction, phobias, and post-traumatic stress disorder.

## 1. Introduction

Organisms, including humans, thrive in complex heterogeneous environments by modifying their behavior, increasing chances of survival and reproduction. Thus, memory is an indispensable mechanism that integrates knowledge and directs future behavior. The integrated information is preserved across different stages in which memory is encoded, integrated, and retrieved (Squire, 2009). Organisms generally recollect information about shelters, food sources, mate recognition and location, and dangerous situations. However, environmental conditions are not fixed, and milieus constantly change; therefore, organisms must adapt their behavior by modifying the previously integrated information. Hence, memory is also a dynamic process that provides flexibility for adaptive response during sustained environmental change. This flexibility enhances survival by updating and editing the integrated information and redirecting behavior according to fluctuating events.

Memory integrates various experiences for different intervals; therefore, memory could be classified depending on the duration and participation of discrete brain structures and circuits, resulting in different memory systems. Memory is classified according to its duration as short-term (STM) and long-term memory (LTM) (Atkinson and Shiffrin, 1968; Norris, 2017). STM concerns the maintenance of information during short periods and involves the covalent modification of existing proteins, temporally changing the strength of pre-existing synaptic connections, while LTM involves persistent morphological and physiological changes yielded by *de novo* protein synthesis facilitating the retention of information for long-lasting periods, even a lifetime (Goelet et al., 1986; McGaugh, 2000; Dudai, 2004; Kandel, 2012). Memory is also classified by the integrated information type and divided into two categories: declarative and non-declarative (Squire, 2004). Non-declarative memory, also named implicit memory, integrates information acquired through repetition, such as habits or motor skills and conditioning (Squire, 2004; Ferbinteanu, 2019). Declarative memory is recalled consciously and subdivided into semantic and episodic memory; semantic memory concerns information associated with facts, whereas episodic memory is related to experienced events (Squire, 2009; Nadel and Hardt, 2011). Episodic memory organizes information associated with “where,” “what,” and “when” an event occurred (Tulving, 2002), facilitating the judgment of whether a recent experience has been previously experienced or encountered and the identification of specific information modalities, including faces, places, sounds, objects, or contextual changes. Recently, emotional components broadened the definition of these classifications, since all these kinds of memories can be integrated under different emotional states, thus enhancing their strength and duration.

**FIGURE 1:**
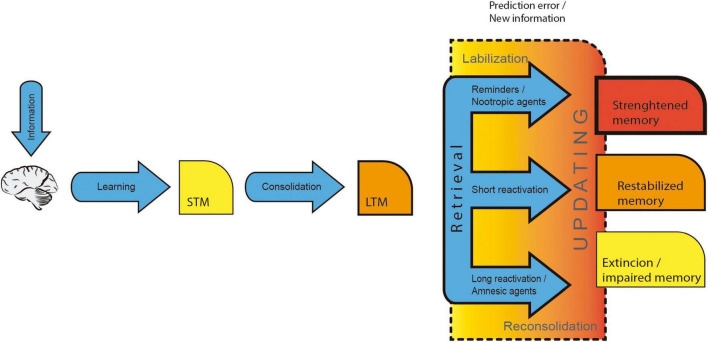
Long-term memory stages. Exteroceptive and interoceptive information is encoded during learning and subsequently stabilized into long-term memory through memory consolidation. Integrated memories are recalled throughout retrieval when they are reactivated and expressed. Furthermore, memory updating occurs only when new information is integrated into the previously formed memory traces.

Memory goes through different stages: encoding, consolidation, retrieval and reconsolidation (Sara, 2000; Abel and Lattal, 2001; Dudai, 2004; Rodriguez-Ortiz and Bermúdez-Rattoni, 2017). Encoding is an attention-dependent process where information is acquired (McGaugh, 2000). Then, information is processed—through protein synthesis—in a time-dependent stabilization mechanism that requires synaptic connectivity modifications within local and systems circuits for LTM integration (McGaugh, 2013, 2000; Bisaz et al., 2014). Memory retrieval refers to the process by which interoceptive and exteroceptive cues select and reactivate integrated information within memory systems resulting in a behavioral outcome (Ben-Yakov et al., 2015; Frankland et al., 2019). After retrieval, LTM can undergo destabilization and restabilization processes conjointly referred to as reconsolidation. Like consolidation, reconsolidation is a time-dependent event that could be affected by amnesic treatments (Nader et al., 2000). Nevertheless, the behavioral response is a dispensable condition during memory retrieval to trigger reconsolidation, since the pharmacological inhibition of memory expression does not affect memory reconsolidation (Rodriguez-Ortiz et al., 2012; Balderas et al., 2013; Santoyo-Zedillo et al., 2014). In this review, we will present evidence suggesting that reconsolidation is initiated every time information is updated, arguing that information updating, and not retrieval, is the crucial factor that triggers the reconsolidation process (Lee et al., 2017; Rodriguez-Ortiz and Bermúdez-Rattoni, 2017). Moreover, reactivated memories can be destabilized after the occurrence of a prediction error when new information is presented concerning previous knowledge. Afterward, LTM goes through a consolidation-like process known as reconsolidation/updating (Nader et al., 2000; Sara, 2000), where memory is enhanced, restabilized, impaired, or modified; it is during this stage that memory updating occurs (see Figure 1; Sara, 2000; Lee et al., 2017; Rodriguez-Ortiz and Bermúdez-Rattoni, 2017). In this work, we will focus on recognition memory editing (Squire and Zola, 1996; Tulving, 2002; Bermúdez-Rattoni, 2004; Balderas et al., 2015; Morici et al., 2015) and valence modification (positive or negative characteristics of the experienced stimulus) (Popik et al., 2020), generating memory updating.

## 2. Updating memory

### 2.1. Recognition and contextual memory

Integrated information within memories is not fixed and is constantly updated because of environmental changes. Declarative and non-declarative memories are susceptible to memory updating and editing; the integrated information predicts the following events. Then, a discrepancy between expectation and reality induces memory destabilization. Declarative memories, like recognition memories, integrate two distinctive processes: familiarity and recollection (Brown and Aggleton, 2001; Merkow et al., 2015). Familiarity conceives whether an event has already been experienced (Mandler, 1980), and the recollection process integrates the event’s specific characteristics (qualitative–valence) (Evans and Wilding, 2012). Recollection is usually associated with the conscious retrieval of the contextual details in which a stimulus occurred (Yonelinas et al., 2010) and requires the integral functionality of several brain structures, including the hippocampal formation and prefrontal, perirhinal, entorhinal, insular, and postrhinal cortices (Brown and Aggleton, 2001; Yonelinas, 2002; Evans and Wilding, 2012; Bermudez-Rattoni, 2014; Merkow et al., 2015). Our understanding of the neurobiological mechanisms related to declarative memory, particularly recognition memory, has been mainly obtained through the evaluation of spontaneous object exploration paradigms. Novel object recognition (NOR) is based on an animal’s innate tendency to explore novel stimuli, where animals discriminate between a previously encoded object and a novel one (familiarity) (Ennaceur, 2010). Another widely employed paradigm is object location memory (OLM). In this task, organisms identify a familiar object in a novel contextual distribution (recollection) (Ennaceur and Delacour, 1988). Both paradigms involve various behavioral sessions; initially, animals are handled and habituated to an empty open field or exploration arena. Then, animals freely explore one or two identical novel objects during the sample phase; throughout the test session, animals are reintroduced to the exploration arena. Recognition memory is assessed either by presenting a different novel object or changing the contextual configuration, NOR and OLM, respectively (Ennaceur and Delacour, 1988; Moreno-Castilla et al., 2018). Novelty demands attention, motivation, and memory processes (Bastin et al., 2019). Thus, NOR alludes that a stimulus has never been encountered (Kafkas and Montaldi, 2018), while an unexpected position/location of the familiar elements is named contextual novelty, as in OLM (Ranganath and Rainer, 2003; Kafkas and Montaldi, 2018; Bastin et al., 2019). NOR (Kelly et al., 2003; Akirav and Maroun, 2006; Rossato et al., 2007; Balderas et al., 2013, 2015; Santoyo-Zedillo et al., 2014) and OLM (Villain et al., 2016; Kwapis et al., 2020; Wright et al., 2020) are susceptible to updating when new information (new object or novel configuration) is presented during reactivation/retrieval and is evaluated in a test session.

### 2.2. NOR and OLM updating

Object-related recognition memory is susceptible to modification and editing. Evidence suggests that NOR memory is only updated when a prediction error occurs. In a NOR updating experiment, animals equally explored two identical objects during the sample phase and then, during the reactivation phase, animals were exposed to different situations. A group of rats explored the same objects as in the sample phase (no prediction error). In contrast, another group explored a new pair of novel objects (totally novel information), and a third group explored a copy of the familiar object with a novel one (prediction error). For OLM updating, a different contextual conformation induces a prediction error. Administration of anisomycin, a protein synthesis inhibitor, within the perirhinal cortex (Balderas et al., 2013) or the hippocampus (Rossato et al., 2007; Choi et al., 2010; Huff et al., 2022) promotes retrograde amnesia, impairing object and contextual memory updating only in the prediction error group. To illustrate this, in an OLM updating protocol, rodents preferred to explore the switched objects due to a novel contextual configuration during the reactivation session. However, if rodents had successfully updated the changed information, they showed a similar preference for all objects, in the test session, when re-exposed to the same contextual configuration, because of contextual familiarity. Nevertheless, administration of anisomycin into the hippocampus impedes memory updating because rodents identify the familiar contextual arrangement as a novel one (Kwapis et al., 2020; Huff et al., 2022). Recognition memory enrolls different structures to update integrated memories depending on the prediction error session. When a prediction error occurs in the expected objects, the perirhinal cortex is mainly involved; however, when the prediction error occurs in the expected context, the perirhinal cortex and the dorsal hippocampus are implicated (Balderas et al., 2008; Winters et al., 2011).

**FIGURE 2:**
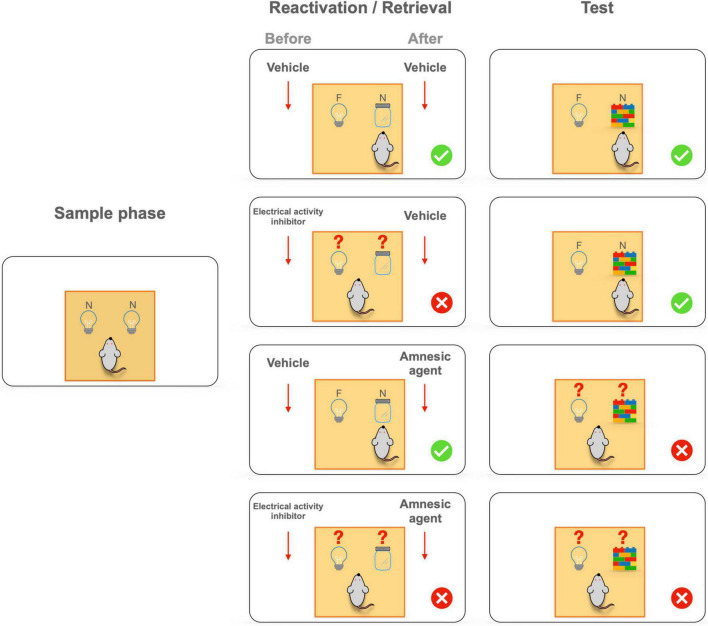
Memory expression is not essential for memory editing and updating. The administration of an expression blocker (a GABA receptor agonist or an AMPA receptor antagonist) before the reactivation/retrieval session impairs recognition memory, since rats could not differentiate between the novel (N) and familiar objects (F). However, rats showed preference for a novel object in the test session, revealing that the original object-related memory remained intact despite inhibition of memory expression. Moreover, the administration of an amnesic agent (a protein synthesis inhibitor) after the reactivation/retrieval session promotes object-related retrograde amnesia, since rats could not differentiate between the familiar and novel objects during the test session. The combined administration of an expression blocker (before reactivation/retrieval session) and an amnesic agent (after reactivation/retrieval session) blunts memory expression during the reactivation/retrieval session and induces object-related amnesia (Based on Balderas et al., 2015).

Therefore, memories are reactivated and destabilized after a prediction error during memory retrieval to integrate updated information. Another characteristic of memory retrieval is the behavioral expression. However, memory expression is not essential for memory editing and updating. The pharmacological inhibition of the perirhinal cortex by the administration of muscimol—a GABA receptor agonist—before the reactivation/retrieval session hinders recognition memory expression, leaving memory destabilization and updating intact (Balderas et al., 2013). Muscimol administration impaired memory expression during the reactivation/retrieval session, since rats had no preference for the novel object, indicating that they could not differentiate between novel and familiar objects. However, in the test session, rats showed preference for a novel object, revealing that the original object-related memory was unimpaired despite the inhibition of memory expression. Moreover, administration of a protein synthesis inhibitor after the reactivation/retrieval session promotes object-related retrograde amnesia, since rats could not differentiate between the familiar and the novel object during the test session. The combined administration of muscimol (before reactivation/retrieval session) and a protein synthesis inhibitor (after reactivation/retrieval session) within the perirhinal cortex inhibits memory expression during the reactivation/retrieval session and induces object-related amnesia (Balderas et al., 2015, 2013; see Figure 2). Likewise, administration of CNQX (before the reactivation/retrieval session), an AMPA receptor antagonist, into the perirhinal cortex interferes with memory expression, observed as a failure to recognize the novel object during the reactivation phase, but maintaining original object-related memory; while the inhibition of N-methyl D-aspartate (NMDA) receptors (after reactivation/retrieval session) with APV or MK-801 leaves NOR expression intact but generates retrograde amnesia (Winters et al., 2009; Santoyo-Zedillo et al., 2014). Conversely, pharmacological blockade of muscarinic receptors or inhibition of protein degradation within the hippocampus prevents destabilization of recognition memory during retrieval, arresting the amnesic effect induced by the administration of a protein synthesis inhibitor stimulus (Choi et al., 2010; Huff et al., 2022). Altogether, these results indicate that NOR destabilization and updating are independent processes from memory expression during retrieval (Figure 2). Although recognition memory updating is usually evaluated by administering amnesic agents, memory updating could also be assessed by its enhancement during the reactivation session. For example, a systemic nicotine administration during the NOR reactivation session promotes better performance during LTM (Tian et al., 2015), indicating that memory strengthening is also a kind of memory updating (Figure 1). Thus, discrepancies between the expected and experienced promote recognition memory destabilization and subsequent integration of the updated information, enabling the editing and modification of existing memories.

Prediction error is commonly associated with dopamine when a discrepancy between the expected and received rewards occurs (Schultz, 2016). However, dopaminergic activity is also involved in other cognitive processes beyond rewards. Dopamine is a modulatory neurotransmitter associated with the regulation of perceptual salience. This memory process modulates the integration of inconspicuous stimuli into a relevant memory facilitating the transition from novelty to familiarity without enhancing the initial sensory perception in recognition memory (Gil-Lievana et al., 2022; Osorio-Gómez et al., 2022). In this regard, the integral functionality of the dopaminergic inputs from the ventral tegmental area and the locus coeruleus is required for novelty detection, comparing the presented information to previously integrated memories (Lisman and Grace, 2005; Lisman et al., 2011). Thus, it has been postulated that dopamine is a general mechanism for predictive processing; this activity signals the prediction error and the difference between the expected value of consequences and the received value (Diederen and Fletcher, 2021). Dopaminergic activity within the hippocampus and insular and perirhinal cortices promotes the consolidation and persistence of familiarity in recognition memory. Howbeit, the evidence related to catecholaminergic activity during NOR updating is limited. D1/D5 receptors pharmacological blockade through administration of SCH 23390 within the hippocampus prevents amnesia caused by the administration of a protein synthesis inhibitor during the reactivation session; these results suggest that D1/D5 receptors are involved in the destabilization process induced by the novel stimulus presented during the reactivation phase (Rossato et al., 2015; Gonzalez et al., 2021). Recently, we demonstrated that optogenetic inhibition of catecholaminergic projections arriving at the dorsal CA1 hippocampus, coming from the locus coeruleus but not from the ventral tegmental area, impedes object location memory updating. Significantly, the pharmacological blockade of hippocampal β-adrenergic receptors with propranolol hinders memory expression without altering memory updating, whereas D1/D5 receptors blockade, by SCH 23390 administration, impairs memory expression and updating (Gálvez-Márquez et al., 2022). These results suggest that dopaminergic activity arising from the locus coeruleus modulates both memory expression and updating when new contextual information is presented. More data are still necessary to comprehend the involvement of dopamine and noradrenaline in the transition of novelty to familiarity in recognition memory. Nevertheless, the gradual transition from novelty to familiarity usually requires several exposures to the novel stimulus, facilitating new information learning (Henson and Gagnepain, 2010) and neural plasticity changes (Lisman et al., 2011). This process suggests that every presentation induces progressive memory updating through reconsolidation processes until complete familiarization is accomplished (Rodriguez-Ortiz et al., 2005).

### 2.3. Taste recognition memory

Novelty detection is crucial since it has been suggested that the novelty-familiarity transition modulates overall recognition memory performance (Parker et al., 1998). Recognition memory is evaluated through different strategies; however, it has also been estimated through evolution-related paradigms, like taste recognition memory, referred to as the ability to identify a particular taste and its relation to post-ingestive consequences (Bermúdez-Rattoni, 2004). Organisms differentiate between novel and familiar food, reducing the ingestion of potentially harmful foods. This behavior is known as taste neophobia; if the tastant stimulus is not associated with positive/negative post-ingestive consequences, the taste becomes familiar, promoting attenuation of neophobia, observed as a gradual augmentation of the stimulus ingestion (Domjan, 1976). Accordingly, novelty detection induces a maximum behavioral response that is gradually diminished after the following presentations, suggesting that taste recognition memory is progressively updated until complete familiarization is accomplished (Rodriguez-Ortiz et al., 2005). Thus, neophobia and its attenuation assess the recognition of memory events necessary to transition from novel to familiar tastes (Osorio-Gómez et al., 2018). Moreover, neophobia and its attenuation are vulnerable to perirhinal and hippocampal lesions (Morillas et al., 2017), like the deficits observed in NOR. This evidence suggests that attenuation of neophobia employs brain structures involved in declarative memories (Moron et al., 2002; Manrique et al., 2009; Grau-Perales et al., 2019).

Another widely used taste recognition paradigm is conditioned taste aversion (CTA). Unlike neophobia and its attenuation, where there are no evident post-ingestive consequences, in CTA, the novel taste is associated with gastric malaise, preventing the animals from consuming the taste in future events (Garcia et al., 1955; Bermúdez-Rattoni, 2004). Hence, aversive taste recognition memory is essential to reject illness-associated tastes. This memory also requires updating and has been evaluated by promoting CTA strengthening through several training sessions (Rodriguez-Ortiz et al., 2012) or changing the expected consequence as in extinction (Garcia-Delatorre et al., 2010) or latent inhibition (Rodriguez-Ortiz et al., 2005). Taste recognition memory comprises two aspects: familiarity and relation to post-ingestive consequences. Therefore, taste recognition memory integrates the information related to the specific characteristics of taste, such as identity, intensity or valence (Breslin, 2013; Wang et al., 2018). Cooperatively, familiarity integrates the information to remember if a taste has been previously experienced. In this regard, results show that novel and familiar stimuli induce the graded activation of several brain regions (Kafkas and Montaldi, 2014). Novel taste exposure promotes catecholaminergic activity within several brain structures (Royet et al., 1983; Dunn and Everitt, 1987; Steketee et al., 1989; Bassareo et al., 2002), including the amygdala (Guzmán-Ramos et al., 2012) and insular cortex (Guzmán-Ramos et al., 2010; Moreno-Castilla et al., 2016; Osorio-Gómez et al., 2021). When the taste becomes familiar, catecholaminergic response is reduced within the nucleus accumbens (De Luca, 2014), amygdala (Osorio-Gómez et al., 2017, 2016), and insular cortex (Osorio-Gómez et al., 2017). Similarly, exposure to a new taste elevates extracellular cholinergic levels within the insular cortex (Miranda et al., 2000; Rodríguez-García and Miranda, 2016); after the taste stimulus becomes familiar, these cholinergic levels decrease and are inversely related to the consumption of the familiar taste stimulus (Miranda et al., 2000).

Consequently, novelty detection induces a maximum response that is gradually diminished after the following presentations, suggesting that attenuation of neophobia can be assessed from a reconsolidation and updating perspective; every time animals are exposed to the taste stimulus, recognition memory is reactivated until complete familiarization is achieved, promoting memory destabilization and facilitating the integration of new information (familiarity) for memory updating (Rodriguez-Ortiz et al., 2005). The administration of a protein synthesis inhibitor into the insular cortex during the initial retrieval sessions of neophobia attenuation hinders memory reconsolidation and updating, generating the familiar taste that is recognized as novel again. However, when the stimulus is familiar, memory is no longer vulnerable to the amnesic effect (Rodriguez-Ortiz et al., 2005). Similarly, administering a muscarinic receptor antagonist within the insular cortex before a second taste familiarization session retards the attenuation of neophobia, and the taste is recognized as novel again (Gutiérrez et al., 2003), impeding memory updating.

Regarding catecholaminergic activity, optogenetic activation of the ventral tegmental area increases the neophobic response. However, optogenetic stimulation of dopaminergic terminals arriving at the insular cortex spares neophobia (Gil-Lievana et al., 2022). Moreover, pharmacological manipulation of the dopaminergic receptors within the nucleus accumbens (shell) or the amygdala impairs taste recognition memory updating. Blockade of D1/D5 receptors in both structures exacerbates the neophobic response even when the stimulus is becoming familiar (second exposure to the stimulus), but attenuation of neophobia is hindered only after the blockade of amygdalar D1/D5 receptors. Nevertheless, activation of D1/D5 receptors within the amygdala diminishes the neophobic response and impedes the attenuation of neophobia updating (Grau-Perales et al., 2020). Therefore, dopaminergic activity requires modulation of the neophobic response and its updating during attenuation of neophobia.

Like object recognition, taste recognition memory updating only occurs when new information is aggregated. Gradual presentation of new information occurs during the novel-familiar transition, but also, novel information is incorporated when the stimulus’ learned characteristics (valence) are changed. In this regard, taste recognition memory is again vulnerable to updating when a familiar stimulus is now associated with post-ingestive consequences, such as gastric malaise, generating a clear taste aversion even after complete attenuation of neophobia has occurred, indicating memory updating (Rodriguez-Ortiz et al., 2005). Inhibition of protein synthesis spares memory updating when the stimulus is familiar since no new information is added. Nevertheless, new information is integrated when the familiar taste is now followed by gastric malaise, making memory vulnerable again to the amnesic effect of protein synthesis inhibition, preventing the incorporation of updated information, i.e., taste aversion (Rodriguez-Ortiz et al., 2005). Taste aversion memory is updated through strengthening. Administration of a protein synthesis inhibitor into the insular cortex or central amygdala impairs aversive memory strengthening during repeated training sessions (García-DeLaTorre et al., 2009). However, when the taste becomes strongly familiar and aversive, due to several conditioning trials, memory is no longer vulnerable to destabilization and memory updating (García-DeLaTorre et al., 2009).

Taste aversion memory updating is an independent process from memory expression. The blockade of D1 dopaminergic receptors within the amygdala spares memory expression but impedes taste aversion updating (Osorio-Gómez et al., 2016). Furthermore, the pharmacological blockade of AMPA receptors within the amygdala (Garcia-Delatorre et al., 2014) impairs conditioned aversive response but spares memory updating, whereas inhibition of protein synthesis (Rodriguez-Ortiz et al., 2012) or the blockade of NMDA (Garcia-Delatorre et al., 2014) within the insular cortex hinders memory updating without interfering with memory expression. In this regard, there is a functional interaction between the amygdala and the insular cortex for taste aversion establishment (Escobar and Bermúdez-Rattoni, 2000; Guzmán-Ramos et al., 2010; Osorio-Gómez et al., 2019) and memory expression and updating (Osorio-Gómez et al., 2017). Through pharmacological manipulations, behavioral analysis, and microdialysis in freely moving rats, we observed that the administration of an AMPA receptor antagonist into the amygdala impairs aversive taste memory expression and prevents norepinephrine and dopamine release within the insular cortex. In contrast, the blockade of NMDA receptors within the amygdala spares aversive taste expression but hinders changes in glutamatergic levels within the insular cortex (Osorio-Gómez et al., 2017). These results suggest that the amygdala modulates memory expression by regulating catecholaminergic activity in the cortex. This was confirmed since blockade of D1 and β-adrenergic receptors within the insular cortex impairs aversive taste memory expression (Osorio-Gómez et al., 2017). However, glutamatergic activity *via* NMDA receptor activation in the amygdala and insular cortex is necessary for memory strengthening through updating (García-DeLaTorre et al., 2009; Garcia-Delatorre et al., 2010; Osorio-Gómez et al., 2016).

Memory updating happens after the appearance of a prediction error, inducing memory destabilization to integrate the new information into the previously formed memory. This process happens during extinction when animals expect that taste will be followed by illness. However, when taste is not followed by gastric malaise, this event promotes memory extinction updating taste information. Inhibition of protein synthesis within the hippocampus or the insular cortex hinders memory extinction since animals still recognize the tastant as aversive, even though the taste is no longer associated with gastric malaise, suggesting that the new information is not integrated into the memory trace (Garcia-Delatorre et al., 2010). Regardless, memory updating induces memory destabilization *via* activation of the ubiquitin-proteasome system; the pharmacological inactivation of this system impairs memory updating, avoiding destabilization and the subsequent integration of new information (Rodriguez-Ortiz et al., 2011). Altogether, if new information is presented during retrieval sessions, memories are destabilized, promoting the integration of the updated information. Taste recognition memory can be updated by familiarizing the taste stimulus when no post-ingestive consequences occur, throughout strengthening memory sessions or when there is a modification in the stimulus’ learned characteristics (valence).

## 3. Emotional valence in memory updating

### 3.1. Integration of interoceptive and exteroceptive information

Several pieces of evidence indicate that the insular cortex translates and integrates external cues into interoceptive states that regulate a broad range of physiological and cognitive processes (Craig, 2009). Consequently, the insular cortex could be postulated as an integrative hub due to the vast reciprocal connections that exist between it and an extensive network of cortical and subcortical structures (Saper, 1982; Craig, 2009; Nguyen et al., 2016; Benarroch, 2019). Thus, as the insular area is responsible for the interoceptive processing of multisensory information, this region could play a vital role in the extensive processing of internal states involved in memory updating (Gu et al., 2013). This hypothesis could be sustained with the established role of the insular cortex in pain processing (Starr et al., 2009; Lu et al., 2016) and negative affective states like anxiety (Paulus and Stein, 2006). According to recent research, the insular cortex participates in mediating several processes related to craving and drug-seeking (Contreras et al., 2007; Naqvi and Bechara, 2009; Moschak et al., 2018) through the upregulation of opioidergic signaling, leading to an altered subcortical function and downstream activity (Pina et al., 2020). Thus, the insular cortex seems to be involved in the integration of multimodal information, including interoceptive and contextual information.

**FIGURE 3:**
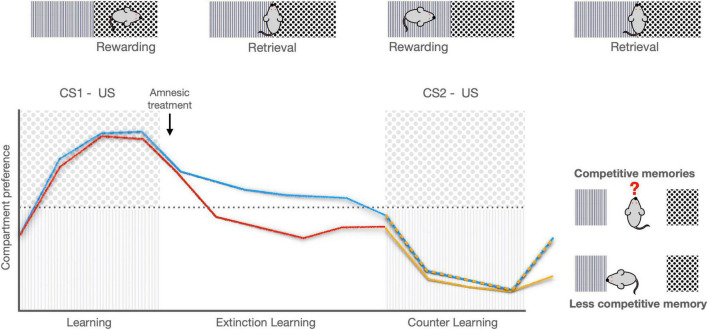
Contextual memory updating. Animals were trained to prefer a compartment (CS1) with a rewarding stimulus (ventral tegmental area photo-stimulation; unconditioned stimulus, US). During the extinction training sessions, control animals are exposed to the conditioned stimulus (CS) without US, initiating extinction learning. The group of treated animals (red) received an amnesic treatment in the insular cortex (NMDA receptor antagonist) and place preference conditioning was extinguished. Then, both groups of animals were counter-trained in the other compartment (CS2) with the same rewarding stimulus. In the retrieval session, the control group maintains the original contextual memory that competes with the new contextual association (blue and orange-dashed line), indicating memory updating. However, the amnesic treatment disrupted the original memory, facilitating the association of new contextual cues with a rewarding stimulus (orange solid line) (Based on Gil-Lievana et al., 2020).

In this regard, contextual information is essential for several learning and memory processes. In a more direct contextual paradigm, the conditioned place preference model, where rodents are trained to associate a rewarding stimulus with contextual cues, memory could be destabilized when mice are re-exposed to the training context without the rewarding stimulus (Milekic et al., 2006; Gil-Lievana et al., 2020). This destabilization makes memory vulnerable to disruption through blockade of NMDA receptors in the insular cortex, inducing amnesia and facilitating the association of new contextual cues with a rewarding stimulus (Gil-Lievana et al., 2020). Interestingly, memory could be re-stabilized when no amnesic agents are given; thus, the original contextual memory is maintained and competes with the new contextual association, even after extinction trials (see Figure 3). Besides, contextual information is gradually incorporated by updating mechanisms that are dependent of protein synthesis. Administration of a protein synthesis inhibitor into the hippocampus impairs memory updating in partially trained animals, whereas the same manipulation in well-trained animals spares spatial memory (Rodriguez-Ortiz et al., 2008). This memory impairment is only observed after new memory encoding at the time of memory destabilization, including memory strengthening, updating or extinction (Morris et al., 2006; Rodriguez-Ortiz et al., 2008).

### 3.2. Salient experiences

During learning, and memory retrieval, specific neural circuits transduce salient experiences (e.g., rewarding and aversive as emotional valence) into instructive neural signals integrated into the memory circuitries (Schultz, 2015). Therefore, salient and emotional experiences processing during learning and memory is a multi-step phenomenon initiated by forming an association between a given stimulus and a related positive or negative consequence every time the stimuli and context are similar. After learning, experience is followed by the development or increase of attention, motivation and/or anticipation, generating a prediction of the event and defined by some as a “state of readiness for a consequence” (Kring and Barch, 2014; Rizvi et al., 2016). Moreover, there is feedback based on consequences and learning during memory retrieval, where a proper sequence of events is required for balanced integration between the expected value of a given stimulus and the predicted consequence (e.g., updating). The consummatory phase of reward or aversive avoidance processing occurs when the goal is achieved, leading to a hedonic (Kring and Barch, 2014; Rizvi et al., 2016) or aversive response (Ozawa and Johansen, 2018).

As expected, several systems that regulate positive or negative valence during emotional/affective processing also interact during associative learning, retrieval and updating. Research of the negative valence role in aversive processing during learning and memory provides insight into the complexity of numerous neurotransmitter pathways that simultaneously impact during aversive vs. hedonic memory. Pharmacological findings demonstrate that noradrenergic activity within the amygdala during aversive and emotional arousal training experiences enhances memory consolidation (Ellis and Kesner, 1983; Liang et al., 1990, 1986; Hatfield and McGaugh, 1999). It is known that aversive experiences produce a surge of noradrenaline in the amygdala (Quirarte et al., 1998; Guzmán-Ramos et al., 2012; Osorio-Gómez et al., 2016). This noradrenergic surge promotes aversive associative learning and memory by activating β-adrenergic receptors (Uematsu et al., 2017). The noradrenergic response arises from the locus coeruleus, which projects to the hippocampus, amygdala and insular cortex, eliciting noradrenaline release (Guzmán-Ramos et al., 2012, 2010; Robertson et al., 2013; McCall et al., 2017; Osorio-Gómez et al., 2021, 2016). Particularly, the noradrenergic modulation of amygdalar activity promotes aversive association since it receives nociceptive information (Bernard et al., 1993, 1992; Bester et al., 1997), improving pain-induced associative learning (Watabe et al., 2013; Han et al., 2015; Sato et al., 2015) and anxiety-related responses (Galvez et al., 1996; Quirarte et al., 1998). Consequently, stress is argued to impact several stages of consolidation and memory updating during complex experiences where an emotional valence induces changes in the allostatic state (e.g., interoceptive and nociceptive modulation) that forms the growing motivational changes in the learned and updated behavior. In healthy humans, the β-adrenergic receptor antagonist propranolol blocks memory reconsolidation in a fear conditioning test (Kindt et al., 2009) and lasts at least 1 month resisting fear reinstatement (Lonergan et al., 2013). Viewing emotional memory updating as a process that includes an allostatic mechanism provides critical insights into how dysregulated neurocircuitry involved in basic motivational systems can transition into pathophysiology. Recent findings (Xue et al., 2017) demonstrate that the administration of propranolol disrupts memory reconsolidation in rats and humans in a nicotine disorder study (Lin et al., 2021). Similarly, propranolol impaired long-term alcohol context-related memory reconsolidation in a rat model (Wouda et al., 2010). Furthermore, some evidence suggests the efficacy of β-blockers in reducing post-traumatic stress disorder (PTSD) symptoms. Thus, blocking memory reconsolidation with propranolol reduced drug addiction and several anxiety/stress disorders (Brunet et al., 2018; Roullet et al., 2021). β-blockers could prevent the associations between environmental stimuli and the effects of self-administered drugs with their respective aversive emotional states. β-blockers decrease the aversive states that include interoceptive nociceptive signals associated with states of anxiety and stress due to the lack of the consumption of substances of abuse (Koob and Schulkin, 2019). Altogether, the evidence indicates the influence of noradrenaline on memory consolidation and memory updating in pathological and salient aversive experiences (Pigeon et al., 2022).

Regarding glutamatergic activity, nociceptive stimuli promote glutamate release, increasing responsiveness, enhancing the aversive response, and inducing the association between nociception and the experienced context (LeDoux, 2000; Bornhövd et al., 2002; Cardinal et al., 2002; Baliki et al., 2006). Furthermore, a recent study reports an increase in calcineurin, an essential plasticity protein, within the basolateral amygdala during fear memory updating (extinction); this protein is modulated *via* NMDA glutamate receptors (Merlo et al., 2014). Consequently, changes in the aversive/negative valence may be related to an increase in glutamatergic activity, through AMPA receptors (Cheng et al., 2011) and NMDA receptor activation, inducing plasticity reeling upon the synthesis of new proteins (Nader et al., 2000) favoring memory updating. Along with it, corticosteroids activate projections from the locus coeruleus to the amygdala, promoting the release of norepinephrine (McCall et al., 2017). Thus, glutamate and norepinephrine modulation of the amygdala enhances aversive memory acquisition and consolidates aversion-related tasks (Roozendaal and McGaugh, 1996; Roozendaal, 2002), and perhaps modulates memory updating. Particularly, noradrenergic and glutamatergic transmission could play an essential role in these pathologies, giving a crucial function to the amygdala-cortical pathways. These findings (see below) suggest that pharmacological intervention in cue-exposure therapies for addictive behaviors and anxiety disorders may be potentiated in understanding the mechanisms involved during new learning, memory retrieval, and memory updating.

Furthermore, emerging evidence gives insights into how acute modulation of opioids can influence memory consolidation and memory updating. Recent reports highlight the importance of the opioid system in regulating not just aversive experiences but also motivation and the sense of hedonic impact (e.g., “*liking,”* the pleasurable/hedonic impact or various expressions of subjective pleasure induced by rewarded appetitive experience) (Peciña and Smith, 2010; Baldo, 2016). In this regard, several neural circuits that are thought to orchestrate feeding behavior overlap with the reward circuitry (Rossi and Stuber, 2018). Some reports agree that opioid peptide neurotransmission causes a shift in the valuation of the “hedonic gradient,” ranging from displeasure to pleasure, which is not limited to the liking of stimuli (Eippert et al., 2008; Haaker et al., 2017). Moreover, micro-stimulation with opioid peptides increases motivation for different cue-triggered seeking responses and innate reward stimuli in rodents (Wassum et al., 2009; Mahler and Berridge, 2012); this data could be linked with growing evidence in animal models and human studies on the involvement of reconsolidation processes in related memories upon their reactivation during relapse to an addictive substance or after traumatic experiences or pathologies.

## 4. Clinical implications of memory updating

Drug addiction and substance abuse disorders are related to the leading causes of mortality and morbidity worldwide (Ritchie and Roser, 2019; Shield et al., 2020; Roser et al., 2021). Some current treatments involve behavioral and pharmacological strategies that acknowledge the psychobiological processes underlying addictions. These can be considered maladaptive reward memories, and the modification or updating of such memories, especially the cue/context reinforcer association, has been addressed through the manipulation of memory reconsolidation (Torregrossa and Taylor, 2013; Liu et al., 2019). Cumulative evidence indicates that propranolol, a β-adrenergic blocker, could be a valuable pharmacological agent to achieve long-lasting results affecting drug-related memories by altering the stability of the memory trace. For instance, in animal models, post-retrieval propranolol administration reduces alcohol-seeking behavior and impairs alcohol-associated memory (Wouda et al., 2010; Schramm et al., 2016). A similar effect was observed with cocaine (Bernardi et al., 2006) and morphine-associated memories (Robinson and Franklin, 2010). In human studies, the administration of propranolol after cocaine cue exposure (memory reconsolidation) decreases craving and physiological responses during a test session. However, this does not indicate memory erasure (Saladin et al., 2013). A small pilot study had similar results over craving severity in patients diagnosed with substance dependence when drug-related memory retrieval took place under propranolol effects (Lonergan et al., 2016). A recent study found a decrease in craving after propranolol reconsolidation disruption in smokers (Lin et al., 2021).

Another process explored to achieve drug-related memory modification is the modulation of the extinction process *via* the glutamatergic system. NMDA receptor agonists (D-serine and D-cycloserine) facilitate the extinction of drug-induced conditioned place preference and reduce reinstatement (Botreau et al., 2006; Myers and Carlezon, 2012; Hammond et al., 2013). In humans, D-cycloserine has been assessed prior to extinction sessions, with poor results in alcohol-dependent subjects and cocaine addicts (Hofmann et al., 2012; Price et al., 2013; Santa Ana et al., 2015) and promising results in smokers (Santa Ana et al., 2009; Kamboj et al., 2012; Otto et al., 2019). Clinical studies have used cue exposure therapy based on the extinction of the conditioned responses elicited by environmental stimuli. The effectiveness of this therapy is limited in a lab-controlled environment (Franken et al., 1999; Marissen et al., 2007; Germeroth et al., 2017), which stresses that the relevance of extinction is mainly context dependent, challenging new therapies to prevent relapse under natural environments.

Emotional memories can be altered through the modulation of integrated information during reconsolidation, opening a possibility for treatment of other types of maladaptive memory traces that trigger undesirable symptoms affecting life quality like the ones associated with PTSD. Propranolol has been assessed as a safe pharmacological strategy to decrease these symptoms (Pigeon et al., 2022). A study reported positive effects after memory reconsolidation under propranolol administration (Brunet et al., 2018). The subjects showed decreased PTSD symptoms under propranolol influence, but other studies failed to produce memory trace destabilization that would allow complete or long-lasting remission (Wood et al., 2015; Roullet et al., 2021). Psychological interventions that aim to disrupt memories during reconsolidation by decreasing the intrusive symptoms have shown some positive effects (Astill Wright et al., 2021); for instance, traumatic memory reconsolidation, a cognitive-behavioral treatment focused on PTSD symptoms, expressed as immediate phobic-like responses triggered by stimuli over a series of treatment sessions where the memory is reactivated and destabilized with a narrative to modify that memory (for details on the treatment see Gray et al., 2019).

Phobias are considered anxiety disorders (American Psychiatric Association [APA], 2013) and are formed by aberrant emotional memories that have a profound and persistent impact on behavior. Different therapeutic approaches have explored the manipulation of memory destabilization-dependent processes (Vaverková et al., 2020). Several reconsolidation-based interventions in animal models of anxiety disorders have successfully used propranolol (Villain et al., 2016). A recent meta-analysis indicated that propranolol administration reduced cue-elicited emotional responses in healthy humans. In contrast, in clinical samples of aversive memories reactivated under propranolol, symptom severity was significantly reduced (Pigeon et al., 2022). This study contrasts with others reporting a lack of post-reactivation propranolol effect on fear of public speaking treatment (Elsey et al., 2020) and arachnophobia (Elsey and Kindt, 2021). Due to the heterogeneity of protocols and environmental conditions of memory reactivation, it has been complicated to reach a clear consensus on the efficacy of propranolol as a treatment tool for any anxiety disorder. A key question is whether the extensive evidence compiled on animal models can be translated as part of a successful treatment of maladaptive memories underlying some psychiatric disorders, given the significant number of confounding factors and limitations.

## 5. Conclusion

Memory editing and updating involve the dynamic and flexible information integration required to thrive under constant environmental alterations. This memory updating modifies the previously integrated information redirecting behavioral response for proper adaptive behavior. Memories are established by consolidation mechanisms that promote morphological and physiological neural changes that subserve memory persistence. Notably, after learning, information integration is accompanied by developing the prediction and expectation of the event and its consequences. Discrepancies between the expected and the experienced promote memory reactivation and destabilization during retrieval, encouraging the integration of new information that adjusts the previously integrated information. Memory updating is necessary for a novel to familiar transition, gradually shifting from displeasure to pleasure, or when a stimulus is no longer followed by a consequence like in extinction trials. Several neurotransmitter systems have been involved in the expression, destabilization, and updating of memories; however, the catecholaminergic system is mainly implicated in memory expression and destabilization, while the glutamatergic system allows the integration of the updated information. After memory destabilization, there is a temporal window where memories are vulnerable to interference. Thus, there is a particular interest in gaining more knowledge about the neurobiological mechanisms involved in destabilization and memory updating. Studying the neurobiological underpinning of memory updating will have potential implications for treating maladaptive memories such as addiction, phobias, and PTSD.

## Author contributions

DO-G, MM, KG-R, and FB-R conceptualized, wrote, reviewed, and edited the manuscript. All authors read and approved the final version of the manuscript.
